# Medical and Educational Indebtedness Among US Health Care Workers

**DOI:** 10.1001/jamahealthforum.2024.1917

**Published:** 2024-07-26

**Authors:** Kathryn E. W. Himmelstein, Alexander C. Tsai

**Affiliations:** 1 Division of Infectious Diseases, Department of Medicine, Massachusetts General Hospital, Boston; 2 Harvard Medical School, Boston, Massachusetts; 3 Center for Global Health and Mongan Institute, Massachusetts General Hospital, Boston; 4 Department of Epidemiology, Harvard T.H. Chan School of Public Health, Boston, Massachusetts

## Abstract

This cross-sectional study evaluates medical and educational debt among the US health care workforce and explores factors associated with higher debt burdens.

## Introduction

Millions of individuals in the US incur educational and medical debts annually, and health care workers may be particularly vulnerable. Extensive training requirements may lead to high student debt among some health care workers, while nonprofessional health workers may be at risk for medical debt due to low wages and poor benefits. Reports have highlighted hospitals’ aggressive debt collection actions, including suing their own employees.^1^

Studies of health care workers’ debts have focused primarily on physician student loan debt.^2^ We evaluated the educational and medical debt of the US health care workforce and factors associated with higher debt burdens.

## Methods

This cross-sectional study analyzed publicly available data from the 2018-2021 Survey of Income and Program Participation (SIPP), a nationally representative longitudinal household panel. The Mass General Brigham institutional review board determined this study was exempt from review. We followed the STROBE reporting guideline.

We compared the medical and educational debts of health care workers (including physicians, registered nurses, nursing aides and assistants, and others; n = 8018, weighted N = 12.6 million) with those of other workers (n = 53 346, weighted N = 83.2 million). To examine the effects of sexism, racism, and other systems of oppression on the distribution of debts, we tabulated the frequency and amount of health care workers’ debts stratified by sex, self-identified race and ethnicity, income, education, occupation, health care industry subsector, unionization status, health insurance, and hospitalization in the previous year. Logistic regression models were fitted to estimate the associations between medical and educational indebtedness and these variables. We used SIPP-provided weights and survey procedures to account for the sample design. Data analyses were performed between April 28, 2023, and May 17, 2024, using SAS, version 9.4 (SAS Institute Inc).

## Results

The weighted sample of health care workers included 76.5% women and 23.5% men; 56.4% were 44 years or younger. Health care workers held more medical debt than other workers (13.9% [95% CI, 13.0%-14.8%] vs 11.1% [95% CI, 10.8%-11.4%]). Mean (SE) medical debt among all health care workers was $1567 ($261), totaling $19.8 billion nationally. Medical debt was more common among female vs male (15.3% [95% CI, 14.2%-16.4%] vs 9.3% [95% CI, 7.7%-10.9%]) and Black compared with White (19.7% [95% CI, 16.8%-22.6%] vs 13.1% [95% CI, 12.0%-14.3%]) health care workers. Medical debt was associated with sex, income, education level, working in home health and nursing home care, lacking health insurance, and recent hospitalization (Table 1).

**Table 1.** Medical Debt Among US Health Care Workers, 2018-2021^a^

Characteristic	Weighted % of all health care workers	Health care workers with any medical debt, % (95% CI)	Medical debt, mean (SE), $^b^	Adjusted OR of holding medical debt (95% CI)^c^
Sex				
Male	23.5	9.3 (7.7-10.9)	1697 (716)	1 [Reference]
Female	76.5	15.3 (14.2-16.4)	1527 (240)	1.36 (1.09-1.70)
Race and ethnicity				
Asian	7.1	4.5 (2.3-6.6)	805 (695)	0.41 (0.23-0.72)
Black	17.0	19.7 (16.8-22.6)	2152 (655)	1.15 (0.93-1.44)
Hispanic	14.3	14.2 (11.9-16.4)	1011 (130)	0.83 (0.66-1.04)
White	58.5	13.1 (12.0-14.3)	1626 (371)	1 [Reference]
Other^d^	3.1	17.0 (11.8-22.3)	1543 (555)	1.18 (0.77-1.81)
Age group, y				
≤44	56.4	14.0 (12.8-15.3)	1270 (312)	1 [Reference]
45-64	36.5	14.4 (12.8-15.9)	2047 (503)	1.10 (0.93-1.31)
≥65	7.1	10.3 (8.0-12.6)	1456 (698)	0.76 (0.57-1.02)
Household income, % of FPL				
<100	4.0	18.1 (13.6-22.6)	2965 (1542)	1 [Reference]
100-199	10.7	22.4 (18.9-25.8)	1233 (384)	1.35 (0.92-1.97)
200-299	13.7	22.2 (19.1-25.3)	2423 (650)	1.64 (1.12-2.41)
300-399	14.8	16.5 (14.0-18.9)	1412 (497)	1.21 (0.82-1.78)
≥400	56.8	9.3 (8.4-10.3)	1364 (402)	0.81 (0.56-1.16)
Education level				
High school or less	24.0	18.8 (16.8-20.8)	1923 (591)	1 [Reference]
Some college	32.9	18.7 (16.9-20.6)	2179 (453)	1.10 (0.91-1.32)
Bachelor’s degree	24.4	9.2 (7.8-10.5)	1183 (623)	0.62 (0.49-0.80)
Graduate degree	18.7	5.5 (4.0-6.9)	537 (349)	0.42 (0.30-0.60)
Occupation				
Nursing aides and assistants	24.2	18.7 (16.7-20.8)	2252 (583)	1 [Reference]
Registered nurses	17.0	12.1 (9.8-14.3)	1956 (809)	1.05 (0.79-1.41)
Physicians	4.7	3.0 (0.6-5.2)	151 (63)	0.62 (0.27-1.46)
Other^e^	54.2	13.3 (11.9-14.7)	1262 (321)	1.10 (0.91-1.34)
Health care industry subsector				
Hospital	36.2	11.1 (9.7-12.6)	1121 (306)	1 [Reference]
Nursing home	16.1	19.7 (17.1-22.3)	2639 (819)	1.41 (1.11-1.81)
Office or clinic	17.9	10.7 (8.8-12.5)	1581 (787)	0.97 (0.74-1.29)
Home health care	10.9	21.8 (18.7-24.9)	1808 (331)	1.65 (1.25-2.18)
Unionization status				
Not union member	88.5	14.2 (13.2-15.1)	1544 (275)	1 [Reference]
Union member	11.5	12.1 (9.5-14.7)	1724 (655)	0.91 (0.68-1.22)
Health insurance status				
Had continuous health insurance	95.9	13.5 (12.6-14.4)	1539 (261)	1 [Reference]
Uninsured for all or part of the year	4.1	23.5 (18.7-28.2)	2206 (1263)	1.56 (1.18-2.07)
Hospitalized during year of analysis				
No	90.6	12.3 (11.4-13.2)	1232 (234)	1 [Reference]
Yes	9.0	30.3 (26.4-34.2)	3550 (681)	2.78 (2.22-3.48)
All health care workers	100	13.9 (13.0-14.8)	1567 (261)	NA

Abbreviations: FPL, federal poverty level; NA, not applicable; OR, odds ratio.

^a^ All dollar amounts are in 2021 dollars (adjusted using the Consumer Price Index). Due to missing data, some percentages may not sum to 100. The CIs were derived from replicate weights using the Fay modified balanced repeated replication method.

^b^ Includes all individuals (with and without debt) in the given subgroup.

^c^ Derived from a single model that included all health care workers and adjusted for sex, race and ethnicity, age, household income, education level, occupation, health care industry subsector, unionization status, health insurance status, and hospitalization during the year of analysis.

^d^ Includes all individuals not identifying as Asian alone, Black alone, or White alone, or as being of Hispanic, Latino, or Spanish origin.

^e^ Includes all individuals working in the health care industry (using Survey of Income and Program Participation industry codes [SIPP]) but not having a SIPP code corresponding to working as a physician, nurse, or aide; this includes phlebotomists, medical records specialists, physical therapists, paramedics and emergency medical technicians, janitors and cleaners who work in a health care setting, and others.

Educational indebtedness was more frequent among health care workers than other workers (26.7% [95% CI, 25.6%-27.9%] vs 16.5% [95% CI, 16.1%-16.9%]). Mean (SE) educational debt among all health care workers was $10 642 ($449), totaling $134.4 billion nationally. Educational debt was more common among Black health care workers than White health care workers and less common among older and lower-income workers and those with lower education levels (Table 2).

**Table 2.** Educational Debt Among US Health Care Workers, 2018-2021^a^

Characteristic	Weighted % of all health care workers	Health care workers with any educational debt, % (95% CI)	Educational debt, mean (SE), $^b^	Adjusted OR of holding educational debt (95% CI)^c^
Sex				
Male	23.5	25.4 (23.2-27.7)	12 550 (1181)	1 [Reference]
Female	76.5	27.1 (25.8-28.5)	10 056 (477)	1.11 (0.96-1.29)
Race and ethnicity				
Asian	7.1	19.7 (16.0-23.4)	9046 (1692)	0.50 (0.39-0.65)
Black	17.0	31.6 (28.3-34.8)	12 650 (1420)	1.57 (1.28-1.92)
Hispanic	14.3	25.0 (22.0-28.0)	6912 (760)	1.02 (0.84-1.24)
White	58.5	26.4 (24.8-28.0)	10 890 (639)	1 [Reference]
Other^d^	3.1	30.9 (24.6-37.3)	15 880 (3682)	1.26 (0.87-1.84)
Age group, y				
≤44	56.4	35.8 (34.0-37.6)	14 246 (730)	1 [Reference]
45-64	36.5	17.0 (15.5-18.5)	6887 (599)	0.35 (0.31-0.41)
≥65	7.1	4.6 (3.0-6.3)	1296 (390)	0.08 (0.05-0.11)
Household income, % of FPL				
<100	4.0	18.7 (13.4-23.6)	7381 (2492)	1 [Reference]
100-199	10.7	23.2 (19.4-27.1)	5991 (784)	1.49 (1.00-2.22)
200-299	13.7	27.4 (24.1-30.7)	9558 (1153)	1.80 (1.23-2.64)
300-399	14.8	27.3 (24.2-30.4)	9034 (874)	1.60 (1.10-2.32)
≥400	56.8	27.6 (26.0-29.3)	12 433 (702)	1.50 (1.05-2.15)
Education level				
High school or less	24.0	6.3 (5.9-6.7)	1819 (300)	1 [Reference]
Some college	32.9	27.9 (25.6-30.1)	5812 (387)	2.99 (2.39-3.73)
Bachelor’s degree	24.4	33.0 (30.5-35.5)	10 447 (709)	4.31 (3.35-5.55)
Graduate degree	18.7	36.7 (33.8-39.7)	30 678 (2006)	6.05 (4.71-7.76)
Occupation				
Nursing aides and assistants	24.2	23.2 (20.6-35.7)	5430 (703)	1 [Reference]
Registered nurses	17.0	34.9 (31.8-38.0)	11 939 (916)	1.11 (0.88-1.40)
Physicians	4.7	28.0 (22.6-33.3	33 141 (4411)	0.75 (0.52-1.09)
Other^e^	54.2	25.7 (24.1-27.2)	10 613 (610)	0.84 (0.70-1.01)
Health care industry subsector				
Hospital	36.2	29.8 (27.8-30.9)	13 657 (962)	1 [Reference]
Nursing home	16.1	22.1 (19.0-25.2)	6375 (990)	0.87 (0.70-1.08)
Office or clinic	17.9	26.4 (23.5-29.3)	11 653 (1356)	0.96 (0.78-1.18)
Home health care	10.9	19.7 (16.4-23.0)	5154 (938)	0.83 (0.64-1.08)
All health care workers	100.0	26.7 (25.6-27.9)	10 642 (449)	NA

Abbreviations: FPL, federal poverty level; NA, not applicable; OR, odds ratio.

^a^ All dollar amounts are in 2021 dollars (adjusted using the Consumer Price Index). Due to missing data, some percentages may not sum to 100. The CIs and SEs were derived from replicate weights using the Fay modified balanced repeated replication method.

^b^ Including all individuals (with and without debt) in the given subgroup.

^c^ Derived from a single model that included all health care workers and was adjusted for sex, race and ethnicity, age, household income, education level, occupation, and health care industry subsector.

^d^ Includes all individuals not identifying as Asian alone, Black alone, or White alone or as being of Hispanic, Latino, or Spanish origin.

^e^ Includes all individuals working in the health care industry (using Survey of Income and Program Participation industry codes [SIPP]) but not having a SIPP code corresponding to working as a physician, nurse, or aide; this includes phlebotomists, medical records specialists, physical therapists, paramedics and emergency medical technicians, janitors and cleaners who work in a health care setting, and others.

## Discussion

US health care workers are more likely than other workers to carry medical and educational debt, collectively owing more than $150 billion. We found that medical debt was more prevalent among women, home health and nursing home personnel, uninsured individuals, and those with recent hospitalization. Educational debts disproportionately burdened Black workers and younger workers and those with higher education.

Educational and medical debts are associated with adverse health outcomes^2,3^ and may limit workers’ professional mobility; reduce workforce diversity; and discourage personnel from entering lower-paying fields, eg, public health or primary care.^2,4,5^ Health care workers indebted to their employers may be less able to address patient safety concerns or protect themselves from workplace abuses.^6^

Self-reported data in the SIPP may be subject to recall bias. The SIPP’s top coding of dollar amounts (which varies by debt type and year) may have led to an underestimation of debt size. No information on the creditor initiating the debt was available, precluding analyses of whether workers were indebted to their employer.

These findings suggest that US health care workers bear substantial educational and medical debts. Further research should assess the effect of such debts on the health care workforce and patient care.

## Notes

Supplement.
Data Sharing Statement
