# Supplementary material for: Medical and Educational Indebtedness Among US Health Care Workers
Source: JAMA Health Forum. 2024 Jul 26;5(7):e241917. doi: 10.1001/jamahealthforum.2024.1917 (PMC11282437; doi:10.1001/jamahealthforum.2024.1917)
Supplement: Supplement. — Data Sharing Statement [file jamahealthforum-e241917-s001.pdf]

Data Sharing Statement

Himmelstein. Medical and Educational Indebtedness Among US Health Care Workers. *JAMA Health Forum*. Published July 26, 2024. doi:10.1001/jamahealthforum.2024.1917

Data
Data available: Yes
Data types: Deidentified participant data
How to access data: SIPP data is publicly available from the US Census Bureau https://www.census.gov/programs-surveys/sipp.html
When available: With publication

Supporting Documents
Document types: None

Additional Information
Who can access the data: SIPP data availability is determined by the US Census Bureau
Types of analyses: SIPP data availability is determined by the US Census Bureau
Mechanisms of data availability: SIPP data is publicly available from the US Census Bureau https://www.census.gov/programs-surveys/sipp.html
